# Pain management in German hospices: a cross-sectional study

**DOI:** 10.1186/s12904-023-01291-5

**Published:** 2024-01-03

**Authors:** Christian Volberg, Henning Schmidt-Semisch, Julian Maul, Jens Nadig, Martin Gschnell

**Affiliations:** 1 Department of Anesthesia and Intensive Care, University Hospital Giessen and Marburg, Philipps University of Marburg, Marburg, Germany; 2 Research Group Medical Ethics, Department of Medicine, Philipps University of Marburg, Marburg, Germany; 3 Institute of Public Health and Nursing Research, Department of Health and Society, University of Bremen, Bremen, Germany; 4 University Children’s Hospital Marburg, Philipps University of Marburg, Marburg, Germany; 5 Department of Dermatology und Allergology, University Hospital Giessen and Marburg, Philipps University of Marburg, Marburg, Germany

**Keywords:** Palliative care, Hospice, Pain management, Pain medication, Palliative sedation

## Abstract

**Background and objectives:**

Pain management is a necessary component of palliative care as most patients suffer from pain during the final phase of life. Due to the complex causation of pain in the last phase of life, it is important to utilize methods other than pharmacotherapeutic options in order to achieve adequate pain control. As little is known about treatment of pain in German hospices, a nationwide survey was conducted.

**Materials and methods:**

All German hospices (259) were contacted by post in June 2020 and asked to participate in an anonymous cross-sectional survey.

**Results:**

A total of 148 (57%) German hospices took part in the survey. A broad variety of medication is used in the hospice setting. Metamizole is the most commonly used non-opiod analgesic , hydromorphone the most commonly used opioid, and pregabalin is the most commonly prescribed co-analgesic drug. The pain medication is usually prescribed as an oral slow-release substance. Standardized treatment schemes are rare among the responding hospices. Most of the respondents also use complementary treatment options, such as aroma (oil) therapy or music therapy, in the treatment of pain. Palliative sedation is used by nearly all responding hospices if all other treatment options fail.

**Conclusion:**

This survey provides an overview of the treatment options for pain management in German hospices. A broad variety of pain medication is used. Compared to international literature, it is debatable whether such a large variety of different types of pain medication is necessary, or whether a reduction in the type of medication available and the use of standardized treatment schemes could benefit everyone involved.

**Supplementary Information:**

The online version contains supplementary material available at 10.1186/s12904-023-01291-5.

## Introduction

As people fear a painful death, adequate pain management is one of the most important goals in palliative care [1]. Pain is particularly common in cancer patients, with a prevalence of 70–80%, and treatment is often not sufficient [2]. In tumor diseases, pain loses its warning and protective function and instead directly restricts patients in their quality of life [3]. Pain is often aggravated by distressing accompanying symptoms (e.g. dyspnea, fatigue, anxiety) and can also be explained by the bio-psycho-social model. According to this model, biological, psychological, social, cultural, spiritual and functional causes play a role in the development and processing of pain [4, 5]. In this context, Cicely Saunders coined the term “total pain”, to describe the experience of pain in the dying process as a complex of physical, emotional, social, and spiritual elements. This severe distress often compromises the bio-psycho-social integrity of the person concerned [6]. Pain in palliative patients should therefore not only be treated using conventional pharmacotherapeutic options. A multi-dimensional and multi-professional treatment concept may thus be helpful [7].

Literature indicates an undertreatment of patients with cancer pain in outpatient palliative care at the beginning of this century [8, 9]. Although the availability of palliative care services has increased over the last twenty years, there is a lack of accompanying studies [10, 11]. Iris Borchmeyer was able to show a significant reduction in pain intensity on the numeric rating scale (NRS) from 6.4 ± 2.87 to 2.5 ± 2.69 after admission to a hospice [12]. In Italy, Elisabetta Petracci and her research group were able to publish similar results in 2016. In the cohort studied, the mean NRS score decreased from 2.58 ± 2.61 to 1.40 ± 1.72 (p = 0.002) within seven days after hospice admission. The decrease was particularly significant in patients who already had more severe pain (NRS ≥ 4) on admission to the hospice. In this subgroup, treatment reduced pain intensity from 5.51 ± 1.24 to 1.76 ± 1.91. They also observed an increase in parenteral administration (e.g. intravenous or subcutaneous) of opioids during the treatment period [13]. Similar results were demonstrated in a review of 2016. It was found that pain management had significantly better outcomes after hospice admission, which also improved quality of life [14]. The analysis of the Swedish health register in 2019 demonstrated that a quarter of the deceased suffered from inadequately treated pain in the last phase of their lives. In this context, statistical analysis revealed that the relative risk of being underserved was significantly higher in hospital than in dedicated palliative care settings [15].

If any form of pain therapy fails and the patient continues to suffer severely, palliative sedation can be initiated as a last resort. The primary aim of this kind of therapy is to control symptoms and relieve stress. In palliative sedation, sedatives are used to induce a sleep-like state that shields the patient from the effects of pain or any other distressing symptom [16–18].

To our knowledge there is no study that fundamentally depicts how pain management is carried out in German hospices. Which drugs and forms of application are used, which professional groups are involved, whether alternative or complementary treatment methods are used and how pain is recorded are currently all unknown factors.

## Materials and methods

A questionnaire with a total of 26 questions was designed. After the positive vote of the local ethics board (file number 19/20; ethics board of the Philipps University of Marburg, Germany) and the registration in the German register of clinical trials (DRKS Reg. No.: 00022360), all German hospices were contacted by post and asked to participate. The necessary addresses were generated via the homepage of the German Association for Palliative Medicine (DGP) [19]. In total, the questionnaire and a prepaid return envelope were sent to 259 hospices in June 2020. The paper-pencil survey was anonymized, and the data analysis was purely descriptive. Data analysis was conducted using Microsoft® Excel, version 16.6.

## Results

Over a period of three months (06/19/2020-09/25/2020) a total of 148 (57.1%) questionnaires were sent back, of which 147 were included in the statistical analysis. The questionnaires were predominantly completed by the hospice management (41%) or nursing service management (37%). Most of the participating hospices can care for between 8 and 12 patients at a time, care exclusively for adult patients (90%) and are not affiliated with a hospital (92%). Further demographic data can be found in Table 1.

**Table 1:** Demographics

Demographics		
**Employee filling the questionnaire**	**Total (n = 147)**	%
Hospice management	60	40.8%
Care management	54	36.7%
Doctor in charge	23	15.7%
Others	10	6.8%
**Beds provided by the hospice**	**Total (n = 147)**	**%**
< 8	10	6.8%
8–10	85	57.8%
11–12	30	20.4%
> 12	22	15.0%
**Care for adults or children**	**Total (n = 147)**	**%**
Adults	133	90.5%
Children	4	2.7%
Both	10	6.8%
**Specialties of the attending physicians**	**Total (n = 146)**	**%**
General practitioner	127	87.0%
Internal medicine	89	61.0%
Anesthesia	62	42.5%
Pediatrics	12	8.2%
Neurology	9	6.2%
Surgery	6	4.1%
Others	64	43.8%
**Affiliated to a hospital**	**Total (n = 146)**	**%**
Yes	11	7.5%
No	135	92.5%
**Cooperation with an outpatient palliative care service**	**Total (n = 145)**	**%**
Yes	103	71.0%
No	42	29.0%

All respondents stated that pain is recorded regularly in their hospice. The numeric rating scale (NRS) is most commonly used to document pain (83%), followed by the visual analogue scale (VAS) (38%) and the verbal rating scale (VRS) (24%). In rare cases, special pain questionnaires are used.

Most hospices (86%) develop individual treatment concepts for patients, while 14% follow an in-house standardized pain management concept. In 89% of the hospices, the WHO analgesic ladder is used as a guideline. The German S3 guideline “Palliative care for patients with incurable cancer” by the German Association for Palliative Medicine is known and used by 55% of the hospices, while 36% stated that they have knowledge of the guideline but do not use it. Only 9% of the respondents stated that they are not aware of the guideline.

### Medication and route of application

**Fig. 1:**
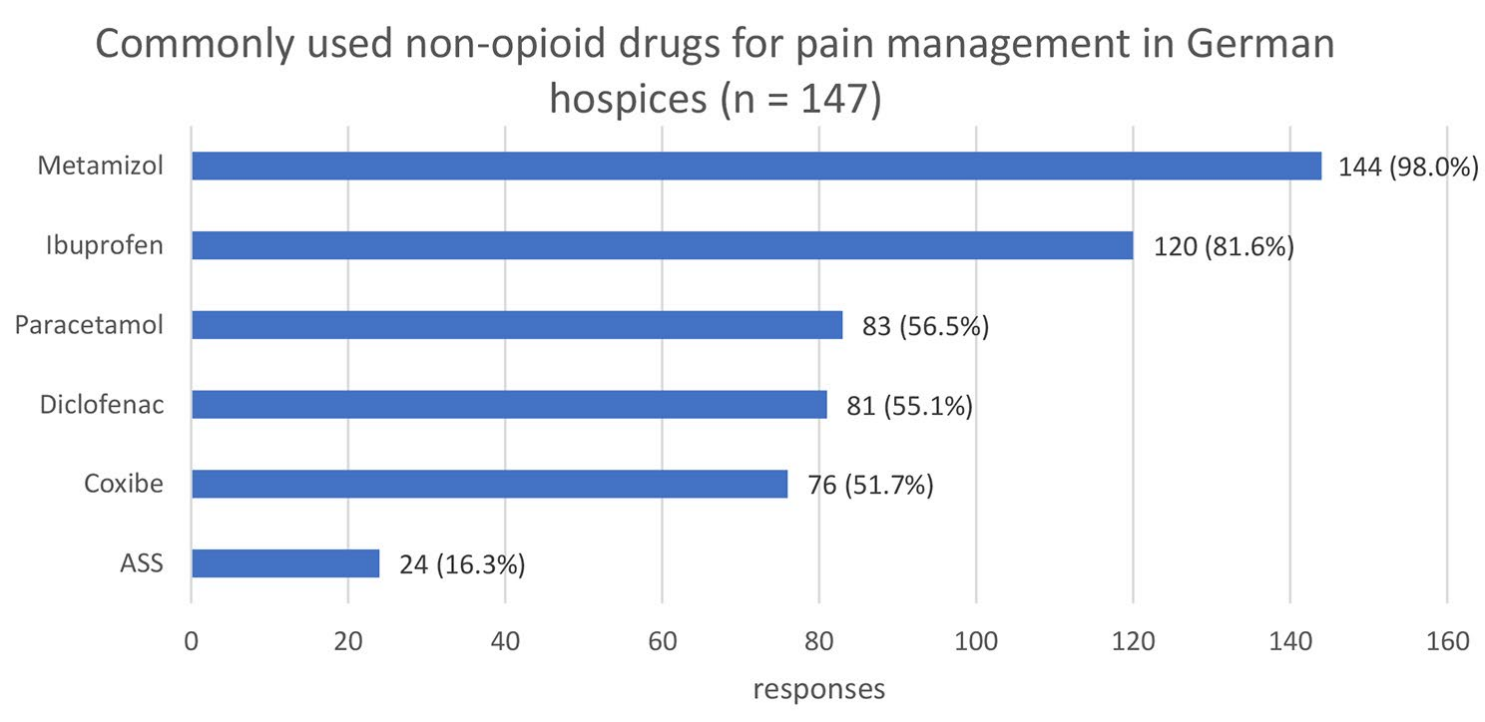
Non-opioid pain medication

**Fig. 2:**
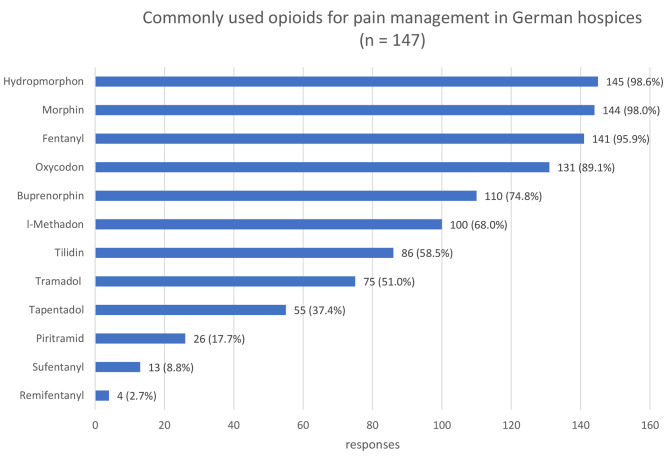
Opioids

**Fig. 3:**
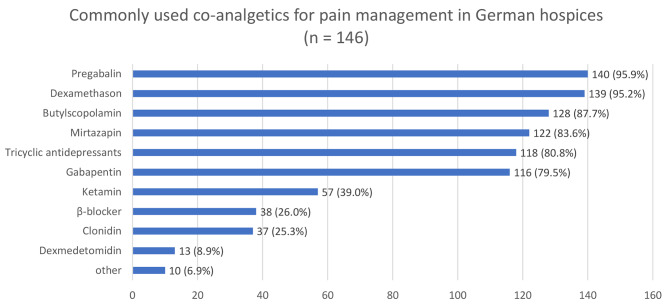
Co-analgesics

The most commonly administered non-opioid analgesics for pain management are metamizole (98%) and ibuprofen (82%) while hydromorphone (99%), morphine (98%) and fentanyl (96%) are the most commonly prescribed opioids. Pregabalin (96%), dexamethasone (95%) and mirtazapine (84%) are the most commonly prescribed co-analgesics (compare Figs. 1, 2 and 3). In most hospices (93%), all pharmaceuticals approved in Germany can be used. Only 4% explicitly stated that they do not offer cannabinoids and 5% do not use methadone derivatives. The majority (87%) stated that they also prescribe laxatives prophylactically.

Opioids are mostly administered orally as a slow-release substance (97%) or as an immediate-release substance (97%), e.g. for breakthrough pain, as well as subcutaneously (97%) (see Table 2). In contrast, intravenous (92%) or subcutaneous (85%) administration is preferred for patient-controlled analgesia (PCA). However, most respondents (79%) stated that PCA is used only occasionally.

**Table 2:** Administration of medication

Form of application	Total (n = 146)	%
Oral long-acting	142	97.3%
Oral short-acting	142	97.3%
Subcutaneous	142	97.3%
Transdermal	140	95.9%
Intravenous	137	93.8%
Buccal	121	82.9%
Nasal	105	71.9%
Others	20	13.7%

Regional anesthesia is not used by the majority (88%) of the participating hospices. Only a small proportion offer peridural catheters (5%), peripheral blocks (3%) or all forms of regional anesthesia (4%). Alternative treatment options are used by 79% of the hospices. Half of the hospices surveyed use aroma (oil) therapy as a complementary form of pain management, and a quarter (24%) use music therapy (including singing bowl therapy). Massages, physical therapy, wraps and compresses, acupressure, psychological care, progressive muscle relaxation are just a few of many supportive forms of treatment offered by hospices (see Table 3). Tumour-specific treatment procedures or invasive pain therapy procedures are not offered by 64% of the participating hospices. Radiation therapy, however, is a treatment option for 28% of the hospices and chemotherapy or immunotherapy is provided by 26% to support pain therapy, for example to reduce the size of the tumour or to inhibit its growth. Invasive pain therapy procedures such as cryoablation or neurolysis are only used by 2%.

**Table 3:** Complementary and alternative therapy options for pain management

Alternative procedures	Total (n = 147)	%
None	30	20.3%
Aroma (oil) therapy	74	50.0%
Music therapy (incl. singing bowl therapy)	36	24.3%
Massage	25	16.9%
Physical therapy (e.g. heat/cold applications)	24	16.2%
Wraps/compresses	21	14.2%
Acupressure (incl. Shiatsu)	15	10.1%
Rubs	14	9.5%
Physiotherapy (e.g. physical exercises)	14	9.5%
Psychosocial support (e.g. talks, attention, comfort)	13	8.8%
Meditation (incl. Yoga)	11	7.4%
Progressive muscle relaxation	11	7.4%
Relaxation exercises	8	5.4%
Homeopathy	7	4.7%
Special positioning	6	4.1%
TENS (transcutaneous electrical nerve stimulation)	6	4.1%
Acupuncture	5	3.4%
Breathing exercises, -therapy	5	3.4%
Animal-assisted therapy	5	3.4%
Hypnosis	4	2.7%
Dream journeys	4	2.7%
Art therapy	4	2.7%
Other (e.g. therapeutic touch, basal stimulation, special washing, complementary care, cranio-sacral therapy)	17	11.5%

### Palliative sedation

Palliative sedation is liberally used by 24% of the responding hospices for patients experiencing severe pain, that cannot be controlled in any other way. In contrast, 73% stated that they only use this option in exceptional cases, while in four hospices (3%) palliative sedation is never used. In addition to pain, other indications for the use of palliative sedation besides pain are dyspnea (78%), “total pain” (75%), terminal agitation (63%) and nausea and vomiting (37%). A combination of morphine and midazolam (84%) is most commonly used for sedation, followed by midazolam (43%) and a combination of morphine and lorazepam (22%) (see Table 4).

**Table 4:** Medication used for palliative sedation

Medication	Total (n = 141)	%
Morphin + Midazolam	118	83.7%
Midazolam mono	60	42.6%
Morphin + Lorazepam	31	22.0%
Morphin mono	23	16.3%
Lorazepam mono	20	14.2%
Ketanest + Midazolam	17	12.1%
Morphin + Propofol	11	7.8%
Propofol mono	7	5.0%
Ketanest mono	6	4.3%
Others	23	16.3%

## Discussion

With a response rate of 57%, the informative value of this anonymous institutional survey can be considered as high. Nevertheless, the comparison with other studies is difficult, as most studies on pain management in hospices have been conducted in the United Kingdom or North America, where the care network for palliative patients is structured differently. In the United Kingdom for example, a hospice (or hospice care) is usually an institution that, in addition to inpatient treatment, also provides outpatient care for patients in their home environment [20, 21]. In Germany, there is a two-level care system for patients suffering from a palliative disease. Primary care is provided by general practitioners, nursing services and hospital physicians at the general palliative care level. The second level includes palliative care units attached to a hospital where acute medical problems can be treated. In addition, there are specialized outpatient palliative care services (German abbreviation: SAPV), which provide palliative care at home, and finally hospices, where people with a low life expectancy can live and receive extensive palliative care until they die [22].

We therefore assume that, due to the lack of comparable surveys, our survey can help to compare different health systems in different countries. Basic research is an important component for the international exchange of research data in the field of palliative care. As we were able to show, in Germany alone there is a wide range of medications and treatment methods used in pain therapy. By showing the different organisational structures and treatment options in different health care systems, advantages and disadvantages in one’s own system can be recognised and compared with each other.

In the retrospective evaluation by Anniek Masman and colleagues, it was found that morphine, midazolam, and haloperidol are among the most frequently used drugs in palliative patients, especially towards the end of life. The administration route of the drugs changes from oral on admission of the patients to intravenous or subcutaneous towards the end of life. They point out that therapy regimes in palliative care are mostly based on experience and lack clear guidelines [23]. In the survey presented here, it was found that only 14% of the participating hospices follow standardized treatment concepts. The majority of respondents create individual pain treatment concepts for their patients, as recommended by the WHO [24]. On the other hand, Patric Bialas was able to demonstrate in this context for inpatient care that the implementation of treatment standards for pain management is associated with positive effects for both patients and practitioners [25]. Whether these effects are transferable to palliative care is yet not known, but at least 14% of the hospices in our survey regularly use treatment standards for pain therapy. This does not mean that there is one medication plan that fits for all patients, but that there are clear guidelines for the escalation of pain therapy that the staff can follow. Further research should focus on the potential benefits. It is also interesting that one third of the respondents stated that they are aware of the national S3-guideline but do not use it in their daily practice. Also, most respondents (89%) stated that they orient their pain management according to the WHO analgesic ladder. In recent years, there has been frequent discussions about whether this scheme in its initially conceived form is outdated and whether other models are needed instead [26, 27]. In general, the analgesic ladder should be used more as a teaching instrument rather than a strict guideline [28]. In this context, Raffa and Pergolizzi presented their idea of the “analgesic pain pyramid” in 2014, which more adequately reflects the different dimensions of pain management [29]. In 2019, the WHO introduced a new guideline for the management of cancer pain in which the analgesic ladder is no longer promoted [24].

Most hospices surveyed describe that they also use alternative and complementary treatment methods for pain therapy (see Table 3). For music therapy in particular, there are some studies that show a benefit [30–32]. Likewise, aroma oil therapy is gaining in importance as a supportive treatment option and was indicated as the most used supportive method in pain therapy in our survey [33, 34]. A surprising result is that one fifth of the surveyed hospices answered that they do not offer alternative treatment methods. WHO recommends psychosocial care as essential component of palliative care and spiritual counselling for the patient and family should be facilitated [24]. Anna Pape et al. were able to show in their Germany wide survey on physical therapy and occupational therapy in palliative care that there are often funding problems or problems finding trained stuff, especially in the outpatient sector [35].

On close examination of the medication used, we found that a large variety of drugs are used. The question is whether such a wide range of medication is useful, or whether treatment of pain could not be managed with a smaller selection of drugs. Studies have shown that side effects and incorrect dosing occur less frequently when practitioners use fewer and therefore more familiar medications [36, 37]. This may also be related to the fact that not every doctor working in palliative care has extensive training in pain management. Various studies have shown that difficulties often exist in the treatment of cancer pain, especially in outpatient care [38–41]. In this context, standardised treatment regimens as described above could be useful and give more security to doctors and nursing staff [25].

Despite optimal palliative care, stressful and uncontrollable suffering can occur at the end of life. This suffering can be triggered by physical, psychological, or existential problems. In these cases, palliative sedation may be indicated to alleviate the suffering [42]. The German Association for Palliative Medicine (DGP) as well as the European Association for Palliative Care (EAPC) point out that palliative sedation is an important treatment option for patients with a high symptom burden [2, 42]. Stephanie Stiel and colleagues highlight a lack of definition and correct use of the term “palliative sedation”, which leads to difficulties in comparing different methods. In their study, they distinguish between low and deep sedation and found that lorazepam, promethazine and (es-)ketamine are the preferred drugs for a deep sedation [43]. Similarly to the work published by Masman et al., morphine and midazolam are also the most commonly used drugs for palliative sedation in our survey [23]. The German “SedPall” project as well as the international “PALSED” project have developed recommendations regarding palliative sedation, to provide advice to practitioners, nurses and relatives on how to deal with this particular form of symptom control. In this context, the EAPC has published a new framework on palliative sedation [42, 44, 45]. The EAPC states that opioids are generally considered inappropriate medications for sedation, so the results of our survey also suggest that there remains ambiguity regarding the definition and proper administration of palliative sedation [42, 46]. Nonetheless, in the context of the present survey on pain management, it is important to consider that the supportive use of opioids may be justified for noncontrollable pain in the setting of palliative sedation. Anne Hopprich also showed in her evaluation that 91% of palliatively sedated patients in a university palliative care unit received an opioid as co-medication for a necessary symptom control of dyspnea or pain [47].

### Limitations

As this is the first nationwide survey on pain management in German hospices, it is difficult to relate the results to other studies because of the varying availability of drugs in other countries (e.g. metamizole) and the different structure of palliative care. Due to the concept of the questionnaire, a systematic bias can be assumed, as the answers represent the attitude and knowledge of the person filling out the questionnaire and not the whole team. The responses may have been influenced by social desirability in some cases, and the hospice management that completed 41% of the questionnaires may not have been familiar with all aspects of the pharmacological pain management. In Germany pharmacological treatment is mostly performed by GPs in the vicinity of the hospice who are not part of the hospice staff but work in general practice. This probably explains why so few physicians participated, and this in turn may have influenced the results. A survey of the entire team would have been desirable but was not possible due to the concept of the survey in its paper-pencil-form. However, with a response rate of 57%, it can be assumed that the answers given apply to the majority of hospices in Germany and therefore reflect the current care situation well. Furthermore, this study cannot make any assumptions about the quality of pain treatment. However, the purpose of the study presented here was to demonstrate which options in pain management (medical as well as non-medical) are available. The results can serve as a basis for upcoming studies.

## Conclusions

This survey is the first of its kind to provide an overview of pain management in the hospice care of palliative patients in Germany. The data show that there is a wide range of possibilities regarding the care of these patients. The management of pain requires multimodal treatment. Different professional groups are involved in the care of seriously ill patients and different strategies, both pharmacological and complementary, are applied. Experience has shown that this combination has a positive effect but is yet to be proven by further research. It should also be investigated whether positive consequences, such as fewer side effects or a higher sense of security among providers and (thus) an improvement in patient care, can be achieved through a reduction of the number of medications offered or by the use of uniform treatment regimens in hospices. Our results indicate that there are still ambiguities regarding palliative sedation. In further research it should be explored why existing guidelines are not known or not applied in many hospices and how acceptance can be improved.

### Electronic supplementary material

Below is the link to the electronic supplementary material.

Supplementary Material 1

## Data Availability

The data can be requested from the corresponding author.

## Declarations

### Authors’ information (optional)

Dr. Christian Volberg, MD, M.A. Palliative Care. Resident in anaesthesia and intensive care medicine at the University Hospital Giessen and Marburg, Marburg site. Research associate in the Research Group Medical Ethics, Philipps University of Marburg. Member of the clinical ethics committee. Member of the German Association for Palliative Medicine (DGP).
