# Supplementary material for: Pain management in German hospices: a cross-sectional study
Source: BMC Palliat Care. 2024 Jan 3;23:7. doi: 10.1186/s12904-023-01291-5 (PMC10763107; doi:10.1186/s12904-023-01291-5)
Supplement: Supplementary file 1 — Supplementary Material 1 [file 12904_2023_1291_MOESM1_ESM.docx]

English translated version of the questionnaire used:

***Pain management in German hospices: A cross-sectional study***

Contact person:

Dr. med. Christian Volberg

University Hospital Marburg - Department of Anaesthesia and Intensive Care Medicine Baldingerstraße - 35043 Marburg

In cooperation with:

Prof. Dr. Gerd Glaeske

SOCIUM Research Centre Inequality and Social Policy Department of Public Health and Drug Application Research Unicom Building - Room 3.4270

Mary-Somerville-Straße 3 - 28359 Bremen

Prof. Dr. Henning Schmidt-Semisch

Institute for Public Health and Nursing Research

Department 6 - Health and Society - Room 0090

Grazer Straße 2 - 28359 Bremen

Pseudonym of the hospice:___________

1. Who completes the questionnaire?

- Responsible medical doctor

- Hospice management

- Head of nursing service / ward manager

- Other:

1. In which federal state is the hospice located?

- The hospice is located in ______________

1. How many beds does the hospice have?

- Our hospice can care for ____ patients

1. Do you care for children or adults?

- Exclusively adult patients

- Exclusively pediatric patients

- Both

1. Is the hospice affiliated to a hospital or independent?

- The hospice is affiliated to a hospital

- The hospice is independent

1. Do you work in cooperation with a specialized outpatient palliative care service?

- No, we work independently

- Yes, we work together with a specialized outpatient palliative care service

1. How many doctors care for the patients in the hospice, work for the hospice or with the hospice in cooperation?

- ____ Doctors are permanently employed for our hospice.

- Our hospice is supported by the doctors of the specialized outpatient palliative care service

- Currently, _____ doctors are responsible for the care of the patients

- Every patient is cared for by his or her family doctor

- As our hospice is affiliated to a hospital, the patients are also cared for by the hospital's doctors

- Other arrangements:

1. What specialties do the doctors in charge come from? (Multiple answers possible)

- General medicine

- Anesthesia

- Internal medicine

- Surgery

- Neurology

- Pediatrics

- Other:

1. What form of documentation of pain is used in your hospice?

- Pain is not documented

- Verbal rating scale (VRS) (no pain, mild, moderate, severe pain)

- Visual analogue scale (pain intensity 0-10)

- Numerical rating scale (pain intensity 0-10)

- Pain questionnaire (e.g., McGill Pain Questionnaire)

- Other:

1. Is there a standardized pain management concept in your hospice or are individual concepts / therapies created for each patient?

- In our hospice, we follow an in-house, standardized pain therapy concept

- Individual pain management concepts are developed for each patient

1. Do you follow the WHO analgesic ladder when setting pain therapy?

- No, we do not use the WHO analgesic ladder

- Yes, we follow the WHO analgesic ladder for pain management

1. Is your hospice aware of the German S3 guideline 'Palliative care for patients with incurable cancer'? Do you follow this guideline for pain management?

- No, the S3 guideline is not known and is not used

- Yes, the S3 guideline is known but pain management is not oriented towards it

- Yes, the S3 guideline is known, and we implement it in our daily work

1. Which application form(s) for pain medication do you use? (multiple answers possible)

- oral long-acting

- oral short-acting

- buccal

- nasal

- transdermal

- s.c.

- i.v.

- Other:

1. What analgesics are used in your hospice? (multiple answers possible)

- Ibuprofen

- Paracetamol

- Metamizole (e.g. Novalgin)

- ASS

- Coxibe (e.g. Dynastat, Arcoxia)

- Diclofenac (e.g. Voltaren)

- Tramadol (e.g. Tramal)

- Tilidine (e.g., Valoron)

- Tapentadol (e.g., Palexia)

- Piritramide (e.g., Dipidolor)

- Morphine (e.g. Sevredol)

- Hydromorphone (e.g. Palladon)

- Oxycodone (e.g. Oxygesic)

- Fentanyl (e.g. Durogesic)

- Sufentanil

- Remifentanyl (e.g . Ultiva)

- Buprenorphine (e.g . Temgesic)

- L- Methadone (e.g . L- Polamidone)

- Other:

1. What co-analgesics are used in your hospice? (multiple answers possible)

- Tricyclic antidepressants, e.g., amitriptyline (e.g., Saroten)

- Gabapentin (e.g., Neurontin)

- Mirtazapine (e.g., Remergil)

- Pregabalin (e.g., Lyrica)

- Ketamine (e.g., Ketanest)

- Clonidine (e.g., Catapressan)

- Dexmedetomidine (e.g., Dexdor)

- β-blockers (e.g., Beloc)

- Dexamethasone (e.g., Fortecortin)

- Butylscopolamine (e.g., Buscopan)

- Other:

1. Do you use patient-controlled analgesia (PCA) systems?

(Patients have, for example, their medication at hand in the room or can give themselves boluses via pump systems)

- No, pain medication is only dispensed by nursing staff

- Yes, occasionally depending on the patient’s compliance

- Yes, as a standard

1. If yes, which application form of PCA do you use? (multiple answers possible)

- intravenous (i.v.) PCA

- subcutaneous (s.c.) PCA

- sublingual (s.l.) PCA

- nasal PCA

- oral PCA

- regional anaesthesia PCA

1. Do you use regional anaesthesia procedures for isolated pain, e.g. pain in one limb or lower abdominal pain?

- No

- Yes, but only peripheral blocks (e.g. femoral or ischial catheter)

- Yes, but only peridural catheters (PDK)

- Yes, all forms of regional anaesthesia can be performed at our hospice

1. Are there alternative or complementary procedures (e.g. hypnosis, meditation, progressive muscle relaxation or similar) that you use for pain management?

- No, we only treat pain with pain medication

- Yes, we use the following procedures as alternatives or as an accompaniment to medication therapy: _______

1. Do you use tumor-specific treatment procedures or invasive pain therapies?

- No, we do not use such therapy concepts

- Radiotherapy

- Chemo-/immunotherapy

- Surgical resections

- Cryoablation

- Neurolysis

- Other:

1. Are there any pain medications that are not used in your hospice?

- No, all medicines approved in Germany can be administered here

- Yes, cannabis or derivatives

- Yes, methadone or derivatives

- Other:

1. Do you give laxatives prophylactically during opioid therapy?

- No, laxatives are only given symptomatically in case of coprostasis

- Yes, every patient receives prophylactic laxatives during opioid therapy to prevent coprostasis

1. In case of uncontrollable pain, do you perform palliative sedation?

- No, palliative sedation is contrary to our principles

- Yes, in exceptional cases we perform palliative sedation

- Yes, palliative sedation for symptom control in cases of uncontrollable pain is carried out by us regularly

1. What other indications does your hospice have for palliative sedation when any other therapy has failed?

- Dyspnoea

- Nausea and vomiting

- Terminal agitation

- Emotional distress, "total pain"

- Other:

1. Which drugs do you use for palliative sedation?

- Morphine mono

- Midazolam mono

- Lorazepam mono

- Propofol mono

- Ketanest mono

- Morphine + Midazolam

- Morphine + Lorazepam

- Morphine + Propofol

- Ketanest + Midazolam

- Other:

1. Do you have any comments? Are there any comments you would like to share with us that were not asked for in the questionnaire? Do you have any suggestions or criticism?

In order to protect the security of your data, this sheet will be separated from the rest of the questionnaire by the trust centre at the SOCIUM Research Centre and will not be included in the evaluation. It is therefore not possible to draw conclusions about your institution during the data analysis (see information letter on data protection).

1. Would you be available for an interview if you had further questions?

- No

- Yes, please contact me as follows:

Surname, First Name:

Address:

Phone:

E-mail:
